# Preventing decompensation among multimorbid outpatients in residential care: a cohort study with a six-month follow-up to prevent decompensation among multimorbid outpatients in residential care

**DOI:** 10.1017/S1463423625100145

**Published:** 2025-08-04

**Authors:** Paul Aujoulat, Jean Yves Le Reste, Lucas Beurton-Couraud, Marie Barais, Benoit Chiron, Pierre Barraine, Morgane Guillou-Landreat, Delphine Le Goff

**Affiliations:** 1 Department of general practice, University of Western Brittany, 22, av. Camille Desmoulins, Brest, FR 29238, France; 2 ER 7479 SPURBO, University of Western Brittany, 22, av. Camille Desmoulins, Brest, FR 29238, France

**Keywords:** Family medicine, multimorbidity, prevention

## Abstract

**Aim:**

The European General Practitioners Research Network (EGPRN) designed and validated a comprehensive definition of multimorbidity using a systematic literature review and qualitative research throughout Europe. This survey assessed which criteria in the EGPRN concept of multimorbidity could detect decompensating patients in residential care within a primary care cohort at a six-month follow-up.

**Method:**

Family physicians included all multimorbid patients encountered in their residential care homes from July to December 2014. Inclusion criteria were those of the EGPRN definition of multimorbidity. Exclusion criteria were patients under legal protection and those unable to complete the 2-year follow-up. Decompensation was defined as the occurrence of death or hospitalization for more than seven days. Statistical analysis was undertaken with uni- and multi-variate analysis at a six-month follow-up using a combination of approaches including both automatic classification and expert decision. A multiple correspondence analysis and a hierarchical clustering on principal components confirmed the consistency of the results. Finally, a logistic regression was performed to identify and quantify risk factors for decompensation.

Findings: About 12 family physicians participated in the study. In the study, 64 patients were analyzed. On analyzing the characteristics of the participants, two statistically significant variables between the two groups (decompensation and Nothing To Report): pain (p = 0.004) and the use of psychotropic drugs (p = 0.019) were highlighted. The final model of the logistic regression showed pain as the main decompensation risk factor.

**Conclusion:**

Action should be taken by the health teams and their physicians to prevent decompensation in patients in residential care who are experiencing pain.

## Introduction

The concept of multimorbidity was first published in 1976 (Brandlmeier, 1976). Multimorbidity has been defined by the World Health Organization (WHO) as people being affected by two or more chronic health conditions (World Health Organization, 2008). Multimorbidity is a very interesting and challenging concept particularly for family medicine (FM), given the increasing prevalence of chronic illness in an aging population across all developed countries. It is closely related to a global or comprehensive view of the patient, which is a core competency of FM, as defined, for instance, by the World Organization of National Colleges, Academies and Academic Associations of General Practitioners/Family Physicians (WONCA) (European Academy of Teachers in General Practice, 2002). It is a global “functional” view (useful for long-term care) versus a “disease” centered point of view (useful for acute care) (Huber et al., 2011).

The European General Practice Research Network (EGPRN) has created a research agenda specifically designed for methodological and instrumental research, which includes the development of primary care epidemiology, focusing on patient-centered health (Hummers-Pradier et al., 2010). A comprehensive definition of the concept of multimorbidity (ie, one which is both understandable and usable for further collaborative research) was an important objective for this research network. The objective was to help researchers in FM to investigate the complexity of patients’ conditions and their overall impact on patients’ health. This concept of multimorbidity could be an additional tool for FPs, enabling them to prevent decompensation (Le Reste et al., 2015a).

A research team, including nine national groups, all active within the EGPRN, has created a research community for the purpose of clarifying the concept of multimorbidity for FM throughout Europe (Le Reste et al., 2012). This group produced a comprehensive definition of the concept of multimorbidity through a systematic review of the literature (Le Reste et al., 2013). This concept was translated into most European languages for use in further collaborative research (Le Reste et al., 2015b). Finally, it was validated using qualitative research throughout Europe (Le Reste et al., 2016) and a specific research agenda was issued (Le Reste et al., 2015b). The EGPRN concept of multimorbidity included a set of different variables assessing patients’ multimorbidity, multimorbidity modulating factors, and multimorbidity consequences.

Decompensation (ie, death or hospitalization in acute care) is a challenge for FM as FPs, being familiar with their patient’s health status (Garrido-Elustondo et al., 2016), could miss tiny factors which, if noticed, could help to prevent decompensation (Lee et al., 2015). A predictive model that could be integrated into their clinical practice could help them to prevent decompensation and avoid serious health outcomes for their patients (Lussier et al., 2016). The EGPRN concept of multimorbidity was considered highly suitable for a purpose such as this. It could lead to a usable model for preventing decompensation.

The French population is aging, with one in three people over 60 years of age in 2050 (Robert-bob, 2006). Consequently, the number of dependent patients, some requiring institutionalization in care homes (CHs), is growing. Patients residing in CHs are included in the EGPRN definition of multimorbidity. In France, as in most European countries, patients in residential care are treated by FPs.

The purpose of this research was to assess which criteria in the EGPRN concept of multimorbidity could detect decompensating patients in residential care within a primary care cohort at a six-month follow-up.

## Materials and methods

The survey was a prospective cohort study.

### Ethics statement

The study was approved by the ethics committee of the University de Bretagne Occidentale. The participants (FPs and patients) provided their written informed consent to participate in the study. The ethics committee approved the consent procedure.

### Research team

Two senior FM researchers, one statistician, and two trainees in FM constituted the research group to improve participant selection and to support the FPs in the inclusion and follow-up procedure. A scientific committee composed of eight European senior researchers in primary care supervised the survey.

### Participant selection

The study population included all multimorbid patients (according to the EGPRN definition of multimorbidity) who met 12 FPs in the Lanmeur residential care home in the county of Finistere (in north-west France). These FPs were drawn from those physicians associated with the Lanmeur residential care home.

Inclusion criteria were patients meeting the criteria for the definition of multimorbidity according to the EGPRN definition: any combination of chronic disease with at least another disease (acute or chronic) or a bio-psychosocial factor (associated or not) or somatic risk factor.

For the team, bio-psychosocial factors meant all psychosocial risk factors, lifestyle, demographics (age, gender), psychological distress, socio-demographic characteristics, aging, beliefs and expectations of patients, physiology, and pathophysiology.

In all cases, monitoring overtime was required, and the patient had to sign an informed consent.

Exclusion criteria were patients not meeting the criteria of the definition of multimorbidity, the inability to follow the study over time, patients on legal protection, and patients for whom survival was estimated at less than three months.

### Data collection

FPs who had agreed to participate worked according to the following plan: first, the multimorbid patient was asked to give his/her consent to participate in the study once the terms had been explained to him/her. After that, FPs completed a questionnaire about their patient.

The purpose of this questionnaire was to explore potential decompensation risk factors within the themes and subthemes of multimorbidity (Table 1).

**Table 1.** Themes and subthemes identified for the multimorbidity concept of EGPRN

Themes	Subthemes
Chronic disease	Chronic condition
	Chronic diseases
	Complexity characteristics of chronic disease
	Psychosomatic diseases
Acute disease	Acute condition
	Acute disease
	Complexity characteristics of acute disease
	Reaction to severe stress and acute disorders
Biopsychosocial factors and somatic risk factors	Demography risk factor
	Lifestyle
	Patient’s beliefs/expectations
	Physiopathology
	Psychological risk factors
	Psychosocial risk factors
	Sociodemographic characteristics
	Somatic risk factors
Coping	Patient’s coping strategies
Burden of diseases	Disease morbidity
	Disease complication
Health care consumption	Use of carers
	Disease management
	Health system
	Health care policy
	Health care
	Health care services
	Malpractice
	Assessment
	Medical history
	Medical procedure
	Pain
	Polypharmacy
	Prevention
	Symptoms/signs/complaints
	Treatment or medication
	Cost of care
Disability	Handicap
	Functional impairments
Quality of life	Health status
	Impairment
	Morbidity implication
	Quality of life
Frailty	Frailty
Social network	Dependence on social network
	Family’s coping strategies
	Social isolation
	Social network
	Support from social network
Health outcomes	Outcomes
	Medical research epidemiology
	Mortality
Core competencies of FP	Holistic approach
	Practical experience of family practitioners with patients
	Family practitioner, as the sole expert in multimorbidity
	Expertise of the family practitioner
	“gut feeling”/intuition
	Person-centered care
	Primary care management
	Specific problem solving skills
Relationship between FP and patient	Communication challenge
	FP’s and patient’s experience

The research group developed this questionnaire according to the definition of multimorbidity. To evaluate the concept of somatic risk factors, the team retained variables around a cardiovascular risk factor, a risk of falling factor, and an assessment of hygiene, nutrition, and physical activity. The risk of falling was calculated using the score CETAF. A first pilot study was used to delete some irrelevant variables: chronic condition (which was already described within chronic disease or psychological risk factor), cost of care (impossible to estimate given the time and resources dedicated to the study), disability (disability/impairment), quality of life and health outcome (that is, the consequences rather than the characteristics of the multimorbidity), physiology (the notion is too broad to be evaluated), demography, and aging (already assessed within sociodemographic characteristics). This questionnaire was accepted by the scientific committee of the research team and tested with FPs and medical students. It was formatted to conform to computerized data collection with the help of EVALANDGO software. Data were saved using Microsoft Excel. Six months after inclusion, FPs were contacted by email to check the status of the patient. The collected data were anonymized and a number was assigned to each patient, in order of inclusion. The patient was then classified into either group according to his/her status: decompensation (D) or nothing to report (NTR). The team defined decompensation, in this context, as the occurrence of hospitalization for more than six days or death during the six months of follow-up. Where necessary, the research team called the FPs to collect these data.

### Data analysis

In order to harmonize the data before statistical analysis, a data cleaning process was completed. Descriptive statistics were used to summarize data and check the data quality. Some missing data were identified. For instance, some questions about the number of specialist consultations per year were unanswered in the case of a few included patients. These missing data had to be detected and to be integrated into the statistical analysis (missing data were replaced by the median value of the group). The biggest recoding work took place during data cleaning: the number of acute and chronic diseases was reconsidered by FP trainees from free text fields completed by FP recruiters. Over one hundred chronic diseases were taken into account. Every change and its rationale was recorded in “a dictionary” which is available on demand from the corresponding author.

Comparisons were made between patients who had decompensated and patients who were in the “nothing to report” group. Continuous variables were compared using the Fisher–Snedecor procedures and the Fisher’s exact test was performed for categorical variables. A multiple correspondence analysis (MCA) was used to reduce the dimensions (factorial method). The patients are represented in factorial space where each dimension represents a combination of the initial variables. As this technique is suitable for qualitative variables only, quantitative variables are not used during the procedure but they are then projected, *a posteriori,* onto the factorial space. Based on the individual coordinates obtained, the technique of hierarchical clustering on principal components (HCPC) was then performed to highlight groups of patients sharing common characteristics. The combination of MCA and HCPC helps in the interpretation of the group results. For the purpose of the evaluation of the optimal number of groups in the dataset and the clustering stability, an alternative procedure was carried out. The same clustering structure was obtained. A clustering quality index (Silhouette) was plotted as suggested by Kaufman (Kaufman & Rousseeuw, 1990). Each cluster was then described and interpreted using a list of qualitative and quantitative variables for which the proportion, or mean of the group, was interpreted in comparison with the global rate or the overall mean.

Finally, a logistic regression was used with the objective of identifying factors associated with the patient’s status at six months. As a particular case of the general linear model, logistic regression is suitable because the dependent variable is binary: D (decompensation) versus (vs) NTR (Nothing to Report). The goal was to find the best subset of variables able to explain the decompensation. In this way, a mixed variable selection procedure, backward–forward, was applied with Akaike information criterion (AIC). The shortcomings of this procedure were minimized by combining the results with expert knowledge. The Wald significance tests were used to strengthen the proposed interpretations and the relevance of the results.

## Results

### Sample

*Participants*

About 64 patients were included. The status at six months was collected for all patients. None was lost to follow-up.

### Data cleaning and recoding

Several non-discriminating variables were removed from the analysis because the entire population included had the same answer: the presence of pharmacological treatment (answer was always yes), material available to the patient (answer was always yes), human aid available to the patient (answer was always yes), institutional life (answer was always yes), unemployment (answer was always no), suicide risk (answer was always no), stress at work (answer was always no), and family history of cardiovascular diseases (answer was always no).

The number of uses of health professionals per year, answer was always 1092 (which is the average number in this nursing home).

### Included patients’ characteristics

**Figure 1.**
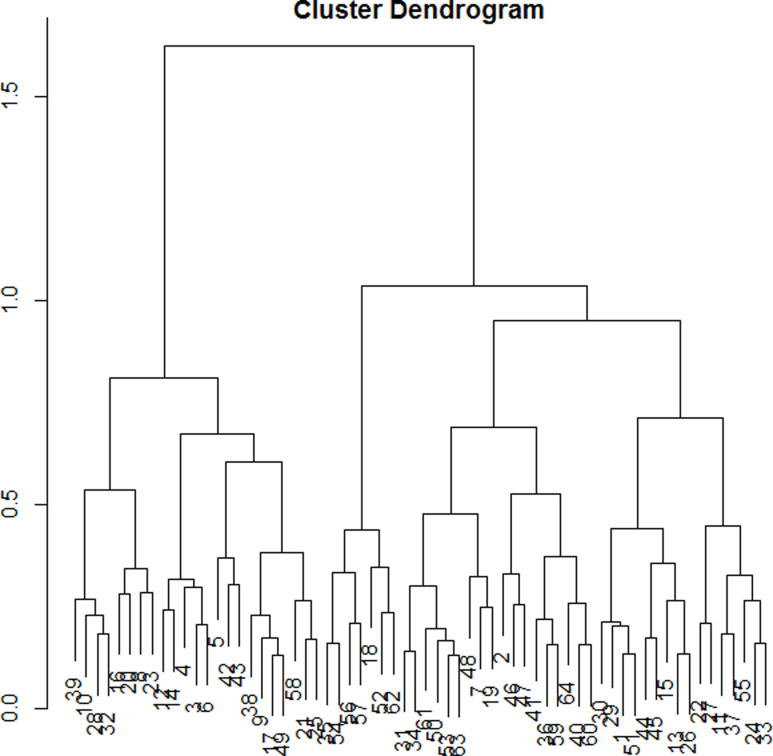
Cluster dendogram.

**Figure 2.**
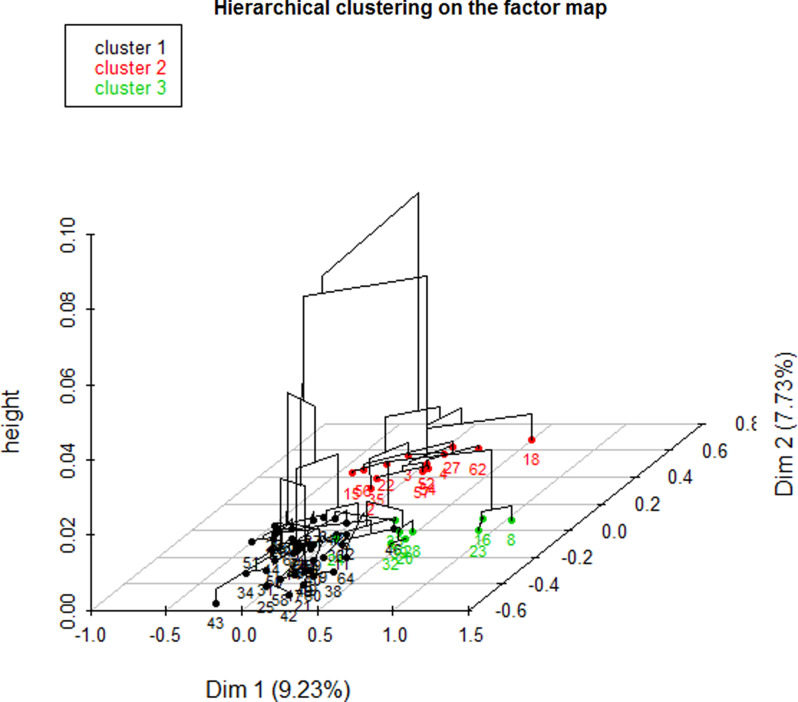
Factor map.

The dendrogram (Figure 1) allowed observation of the hierarchical groups formed from the aggregation of individuals during the hierarchical clustering. The height of a branch is proportionate to the distance between individuals or groups of individuals. Graphically, the dendrogram suggested between 2 and 5 groups, whereas a clustering quality index was maximized for 3 groups of patients. After discussion among the group of experts, the model with 3 clusters was also found to be the most meaningful from a clinical perspective. Figure 2 depicts the 3 groups of individuals projected on the MCA factor map (first two dimensions).

In order to interpret each patient group, a comparison of the proportion within the group (PwG) and within the study population (PwP), meaning the 64 patients, allowed the team to understand the importance of a variable in measuring specific characteristics.

**CLUSTER 1**

In this cluster, all patients were single (PwG: 100% vs. PwP: 19%) and without any psychological risk factor (PwG: 100% vs. PwP: 84%), and none showed addictive behavior (PwG: 100% vs. PwP: 87%) or risky lifestyle (PwG: 100% vs. PwP: 95%). Usually, they accepted screening (PwG: 26% vs. PwP: 19%) when suggested. Most of them had experienced the loss of a close relative (PwG: 86% vs. PwP: 69%) or had at least one dependent relative (PwG: 90% vs. PwP: 83%). Often, the patients were female (PwG: 76% vs. PwP: 62%), and a large majority had postural instability (PwG: 86% vs. PwP: 73%). This cluster is characterized by patients without a reaction to severe stress (PwG: 52% vs. PwP: 42%) and without a somatic risk factor (PwG: 90% vs. PwP: 81%).

**CLUSTER 2**

A large majority of patients in this group had been living as a couple or were widowed (PwG: 92% vs. PwP: 81%). Most of them had not experienced the loss of a close relative (PwG: 77% vs. PwP: 31%). Often, this group was associated with a lower proportion of patients with chronic pathologies (PwG: 61.5% vs. PwP: 34.4%). None of the patients refused screening (PwG: 100% vs. PwP: 81%) when offered.

**CLUSTER 3**

All patients in this group had at least one psychological risk factor (PwG: 100% vs. PwP: 84%). For example, most of them had an addiction (PwG: 89% vs. PwP: 12.5%) or immunodepression as a somatic risk factor (PwG: 67% vs. PwP: 19%). Relatives did not develop any adaptive strategies to cope with the patient’s situation (PwG: 100% vs. PwP: 64%).

### Included patients’ characteristics and status at six months

Six months after inclusion, the population was divided into two groups: 12 “decompensation (D)” and 52 “nothing to report (NTR).”

In the decompensation, group 9 patients were dead and three patients had been hospitalized for more than six days.

Characteristics of each group are reported in Table 2.

**Table 2.** Characteristics of the study population

	Study population *n* = 64 (%)	D *n* = 12 (%)	NTR *n* = 52 (%)	*P*-value
Men	24 (37.5%)	4 (33.3%)	20 (38.4%)	1
Women	40 (62.5%)	8 (66.7%)	32 (61.5%)	1
Average age (mean +/− SD)	84.75 (4.6)	86 (3)	84.46 (4.4)	0.002
Number of chronic conditions	5.7	5.75	5.65	0.723
Hypertension	47 (73.4%)	10 (83.3%)	37 (71.1%)	0.490
Hypercholesterolemia	10 (15.6%)	1 (8.3%)	9 (17.3%)	0.331
Diabetes	7 (10.9%)	0 (0%)	7 (13.4%)	0.293
Osteo-articular disease	42 (65.6%)	9 (75%)	33 (63.4%)	0.521
Psychosomatic disease	18 (28.1%)	5 (41. 7%)	13 (25%)	0.293
Complexity of chronic disease	42 (65.6%)	6 (50%)	36 (69.2%)	0.312
Number of acute conditions	0.2812	0.3333	0.2692	0.611
Reaction to severe stress	37 (57.8%)	9 (75%)	28 (53.8%)	0.213
Complication of acute disease	24 (37.5%)	4 (33. 3%)	20 (38.4%)	1
Overweight	24 (37.5%)	6 (50%)	18 (34.6%)	0.341
Immune-depression	12 (18.7%)	2 (16. 7%)	10 (19.2%)	1
Postural instability	47 (73.4%)	8 (66. 7%)	39 (75%)	0.718
Number of falls over a one-year period	26 (40.6%)	5 (41.7%)	21 (40.3%)	1
Risk behavior	3 (4.7%)	1 (8.3%)	2 (3.8%)	0,470
Average number of falls in a year	0.83	0.83	0.83	0.696
Addiction	8 (12.5%)	1 (8. 3%)	7 (13.4%)	1
Marital problems	1 (1.5%)	0 (0%)	1 (1.9%)	1
Family problems	6 (9.3%)	1 (8. 3%)	5 (9.6%)	1
Financial and social insecurity	11 (17.1%)	3 (25%)	8 (15.3%)	0.418
Death of one or more relatives	44 (68.7%)	7 (58. 3%)	37 (71.1%)	0.493
Divorce	1 (1.5%)	1 (8. 3%)	0 (0%)	0.188
Good hygiene	24 (37.5%)	3 (25%)	21 (40.3%)	0.510
Physical activity	4 (6.2%)	0 (0%)	4 (7.6%)	1
Healthy diet	32 (50%)	5 (41. 7%)	27 (51.9%)	0.750
Children	49 (76.5%)	9 (75%)	40 (76.9%)	1
Coping strategy	28 (43.7%)	8 (66. 7%)	20 (38.4%)	0.108
Complication of chronic disease	28 (43.7%)	6 (50%)	22 (42.3%)	0.750
Number of FP consultations per year	18.08	20.42	17.54	<0.001
Number of specialist consultations per year	1.344	1.417	1.327	0.527
Treatment with risks	26 (40.6%)	3 (25%)	23 (44.2%)	0.331
Daily use of psychotropic medication	60 (93.7%)	9 (75%)	51 (98.1%)	0.019
Coordination procedure	40 (62.5%)	8 (66.7%)	32 (61.5%)	1
Number of biological tests per year	4.703	4.833	4.673 (%)	0.548
Number of medical imaging tests per year	0.7656	0.8333	0.75 (%)	0.640
Good communication between carers	56 (87.5%)	10 (83. 3%)	46 (88.4%)	0.637
Negligence toward the patient	7 (10.9%)	1 (8. 3%)	6 (11.5%)	1
Patient victim of iatrogenesis	18 (28.1%)	2 (16. 7%)	16 (30.7%)	0.483
Lack of time or remuneration	18 (28.1%)	2 (16. 7%)	16 (30.7%)	0.483
Serious and complex medical history	46 (71.8%)	8 (66. 7%)	38 (73.1%)	0.726
Vaccination recommended	63 (98.4%)	12 (100%)	51 (98.1%)	1
Screening proposed	7 (10.9%)	2 (16. 7%)	5 (9.6%)	0.607
Screening accepted	12 (18.7%)	2 (16.7%)	10 (19.2%)	1
Therapeutic patient education proposed	3 (4.6%)	0 (0%)	3 (5.7%)	1
Pain	19 (29.6%)	8 (66. 7%)	11 (21.1%)	0.004
Multiple complaints	17 (26.5%)	4 (33. 3%)	13 (25%)	0.718
Supporting entourage	52 (81.2%)	11 (91. 7%)	41 (78.8%)	0.436
Knowledge about the health system	47 (73.4%)	9 (75%)	38 (73.1%)	1
Problems of habitual, complex behavior	59 (92.1%)	11 (91. 7%)	48 (92.3%)	1
Overview of diseases	61 (95.3%)	11 (91.7%)	50 (96.1%)	0.470
Person-centered care	60 (93.7%)	11 (91. 7%)	49 (94.2%)	0.574
Long-term relationship	60 (93.7%)	11 (91. 7%)	49 (94.2%)	0.574
Intuition	35 (54.6%)	8 (66.7%)	27 (51.9%)	0.522
Quality communication	58 (90.6%)	10 (83.3%)	48 (92.3%)	0.313
Multimorbidity influence on care quality	49 (76.5%)	10 (83.3%)	39 (75%)	0.715

Two qualitative variables compared by the Fisher test were significant (p < 0.05):

- Patients of “D” group suffered significantly more pain than group “NTR”: 67% in the “decompensation” group compared with 21% in the group “NTR” (p = 0.004).
- Patients of “D” group used significantly less psychotropic medication (75%) than group “NTR” (98%) (p = 0.019).

A variance test was achieved for quantitative variables. Two means were statistically different between the groups: age (p = 0.002) and the number of FP consultations per year (p < 0.001).

### Logistic Regression: Research for decompensation risk factor

**Figure 3.**
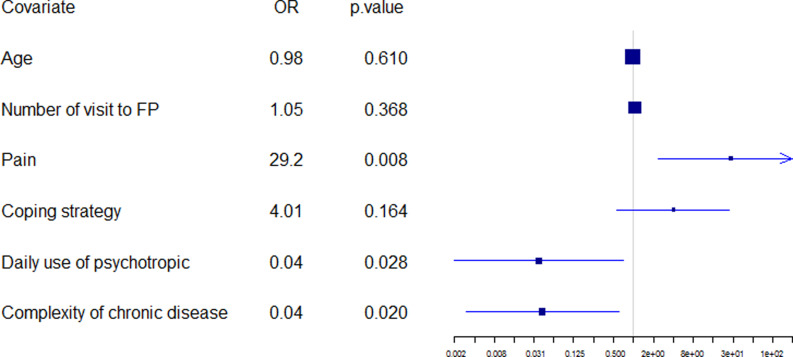
Final regression model.

Logistic regression was performed according to the backward–forward procedure used with the AIC to select the most relevant variables. The initial model included all variables with the removal of those which were non-discriminatory. An automatic selection contained 51 variables with substantial “standard error.” It was necessary to bring expert knowledge to bear, at this level in the automatic scanning, in order to reduce the number of variables produced. For example, the names of chronic diseases were removed (bone and joint disease, diabetes, high cholesterol, high blood pressure…), favoring a number of chronic diseases (and not their type as the list was not extensive). Then, the second initial model included 39 variables (available on demand from the corresponding author). An automatic selection contained 12 variables and the final model (figure 3) was obtained using expert knowledge to select the most significant variables and associated confounding factors (which were not necessarily significant, such as age). As the complexity of chronic disease was borderline significant, the research group wished to check up on antecedents and coping strategies. The first was not significant, once adjusted, whereas the second, coping strategy, was close to being significant, at the 90% level.

Three variables were statistically significant: Patients suffering pain (OR, 29.2; p = 0.008), complex characteristics of chronic disease (OR, 0.04; p = 0.020), and daily use of psychotropic medication (OR, 0.01; p = 0.028).

### Key points

The most relevant information from the final regression model was as follows:

- 40% of patients suffering pain had decompensated over the six-month period (p = 0.012);
- 20% of the pain-free patients who presented no complex characteristic of chronic disease had decompensated during the same period;
- A pain-free patient with a complex characteristic of his chronic disease had no added risk of decompensation; and
- A pain-free patient with a daily use of psychotropic medication had no added risk of decompensation.

## Discussion

### Main results

The description of the population showed 3 statistically different features between the “D” group and the “NTR” group: pain (which was higher in the “D” group), daily consumption of psychotropic drugs (which was less in the “D” group), and a complex characteristic of chronic diseases (which was less in the “D” group).

Pain was a factor that appeared in the description of the population but also in the final model. The difference was very statistically significant between the two groups and remained significant after adjustment: patients of the “D” group suffered significantly (p = 0.008) more pain than the “NTR” group: 67% in the “D” group compared with 21% in the “NTR” group. Many studies focused on the prevalence of pain in CHs (Smalbrugge et al., 2007; Fox et al., 1999) and the impact of pain on quality of life (Hopman-Rock et al., 1997), but the impact of pain on the decompensation of the elderly had not been studied. The studies of analgesia procedures suggested that proper management of pain was a factor that reduced the length of hospital stays (Vaishya et al., 2015; Essving et al., 2011). Pain in the elderly was a common and well-known phenomenon with several rating scales: Self-evaluation (digital scale EN or verbal scale EVS) and hetero-evaluation of chronic pain (by DOLOPLUS® or ECPA) or acute pain (by ALGOPLUS). Three significant factors could contribute to this: lack of proper pain assessment, potential risks of pharmacotherapy in the elderly, and misconceptions regarding both the efficacy of non-pharmacological pain management strategies and the attitudes of the elderly toward such treatments (Gagliese & Melzack, 1997).

Daily use of psychotropic drugs was a protective factor. This could seem inconsistent as it was admitted that psychotropic molecules, such as benzodiazepine, increased the risk of side effects in the elderly (including falling and confusion) and therefore, the risk of hospitalization or death. Nevertheless, health teams were aware of the importance of following up their patients on psychotropic drugs, and we could hypothesize that this could lower the follow-up of those without these drugs.

The final model showed a link between decompensation and the absence of any complex characteristic of chronic disease. This variable referred to the burden of disease. The variable « number of diseases » did not emerge from the analysis as opposed to the variable « complex characteristic of chronic disease » while many previous studies on multimorbidity did not highlight the importance of the « burden of disease » and put diseases of very different severity on the same level (Fortin et al., 2012). The result of the final model seemed to go against logic: in fact, patients who did not present any « complex characteristic of chronic disease » were more at risk of decompensation. We could hypothesize that the presence of a « complex characteristic of chronic disease » generated more stringent monitoring by the health team and thus a decreased risk of hospitalization or death.

Strengths and weaknesses

#### Selection bias

One of the exclusion criteria was an estimated survival expectation of less than three months, this estimate was based solely on the feeling of the team and therefore brought an element of subjectivity to inclusion (Colleter, 2015).

The characteristics of the study population differed from those of the population of French CHs. This was due to recruitment centered solely on the Lanmeur CHs (Colleter, 2015).

#### Information bias

During the analysis, some results had appeared inconsistent:

- No patient had a cardiovascular antecedent. This is probably related to the fact that « antecedents » were not well documented in the files.
- The annual number of demands for paramedic attention was chosen arbitrarily at

three per day because it was too difficult to assess in a CHs.

- Two patients were noted to have agreed to screening when this had not been prescribed for them. These questions should be changed to “Did the patient receive personal or organized screening?”
- The patient’s pain was the theme that presented the greatest difference when compared with the French CHs population as a whole (29.69 % in Lanmeur versus 71.50 % in mainland France as a whole) (Colleter, 2015). This was probably due to the information collection method: the question was put to the FP who responded according to his or her own memory of the patients’ conditions (probably remembering the patients with severe pain but not necessarily those with moderate pain). A standardized scale to measure pain was not used. Indeed, many studies on the subject showed a high prevalence of pain in CHs. For example, a Dutch study of 350 CH patients « Pain-prevalence was 68.0% (40.5% with mild pain symptoms, 27.5% with serious pain symptoms) » (Smalbrugge et al., 2007). In another example, in 1999, a systematic review found that the prevalence of pain ranged from 49% to 83% in CHs (Fox et al., 1999). One hypothesis could be that FPs in this study only focused on serious pain as the level of consumption of painkillers in the Lanmeur CH is the same as in other French CHs.

#### Confounding bias

The themes and sub-themes chosen as references were in English. The questionnaire was conducted by translating from English into French and transforming qualitative into quantitative values. Some translations or transformation values could be inaccurate.

This pilot study was performed using the most appropriate statistical method. However, the small number of patients compared to the large number of variables was the source of many difficulties throughout the analysis.

The choice was made to reduce the number of variables: variables that did not seem relevant statistically, or according to expert knowledge, were removed from the analysis. The results of the final model obtained depended on the choices made previously. These results could have been different if the choices made during the analysis had been different.

### What is new with the results

Many studies have been conducted to assess the relationship between multimorbidity and health outcomes but without solving the problem of the meaning or the intensity of that relationship (Winograd, 1991; Speechley & Tinetti, 1991). The most effective variables identified by this study simplify the concept when it targets the specific outcome of decompensation. This is helpful for clinicians in everyday practice. It could resolve the debate around measuring a concept as broad as multimorbidity appears to be.

Focusing on the number and description of all active chronic diseases seemed pointless. Much of the multimorbidity prevalence research used a list of chronic diseases (van den Bussche et al., 2011; Fortin et al., 2006; Wicke et al., 2014). However, those lists of chronic diseases were very different. In addition, the criteria for the selection of the diseases were often pragmatic (Diederichs et al., 2011). Based on this observation, the team chose to consider almost all chronic diseases listed by the FPs. However, in the end, this was not important for predicting decompensation. A massive amount of research aimed at clusters of diseases now seems inefficient in predicting decompensation.

### Implications for practice, teaching, and future research

In practice, pain should alert FPs and geriatrists to the possibility of decompensation in a multimorbid CHs patient in the subsequent six-month period.

In teaching activities, trainees should be made aware of the importance of this “risk factor.”

In future research, these results should be confirmed by a large-scale study. This study is included in an EGPRN project which aims to define the best possible intervention in order to prevent decompensation in multimorbid patients within 11 European countries.

## Conclusion

The pain seemed to be decisive in determining decompensation in multimorbid patients in residential care. The number of chronic diseases and the burden of these diseases do not make any difference. Preventing decompensation, by using the EGPRN concept of multimorbidity, has now become more straightforward in everyday practice within residential CHs.

## Data Availability

Data supporting the findings of this study are available from the corresponding author [JYLR].
